# A diagnostic challenge: unveiling chronic thromboembolic pulmonary hypertension in the absence of visible thrombi: a case report

**DOI:** 10.1093/ehjcr/ytag034

**Published:** 2026-01-28

**Authors:** Hoang Phu Quy, Dung Doan Duc, Thang Nguyen Duc

**Affiliations:** College of Health Sciences, VinUniversity, Vinhomes Ocean Park, Gia Lam District, 100000 Hanoi, Viet Nam; Cardiovascular Center, Vinmec Times City, 458 Minh Khai Street, Vinh Tuy Ward, Hai Ba Trung District, 100000 Hanoi, Viet Nam; College of Health Sciences, VinUniversity, Vinhomes Ocean Park, Gia Lam District, 100000 Hanoi, Viet Nam

**Keywords:** Chronic thromboembolic pulmonary hypertension (CTEPH), Heart failure with preserved ejection fraction (HFpEF), V/Q mismatch, CTPA, Diagnostic challenge, Case report

## Abstract

**Background:**

Chronic thromboembolic pulmonary hypertension (CTEPH) is a severe condition characterized by persistent dyspnoea and hypoxia. Diagnosis is challenging, especially when computed tomography pulmonary angiography (CTPA) shows no visible thrombi and symptoms overlap with common conditions like heart failure.

**Case summary:**

We present a 77-year-old female with heart failure with preserved ejection fraction who experienced progressive, refractory dyspnoea and hypoxia. Despite a CTPA showing no large, organized thrombi, a strong clinical suspicion prompted a ventilation/perfusion (V/Q) scan. The scan revealed multiple mismatched perfusion defects, confirming CTEPH. A workup for an incidental right ventricular thrombus did not reveal an underlying prothrombotic state.

**Discussion:**

This case highlights the diagnostic difficulties of CTEPH when confounded by comorbidities. Persistent, unexplained hypoxia should trigger a comprehensive evaluation that includes a V/Q scan, which remains the gold standard screening tool due to its high sensitivity. The management approach for inoperable patients requires careful consideration of approved medical therapies and patient-specific factors.

Learning points

- CTEPH can occur without visible thrombi on CTPA; V/Q scanning remains the gold standard for definitive diagnosis in patients with unexplained pulmonary hypertension.
- Persistent dyspnoea in heart failure patients warrants comprehensive evaluation including V/Q scanning, especially when initial imaging is unrevealing.

## Introduction

Chronic thromboembolic pulmonary hypertension (CTEPH) is a distinct form of pulmonary hypertension caused by unresolved thromboemboli, leading to increased pulmonary vascular resistance and progressive right heart failure.^1^ Unlike other forms of pulmonary hypertension, CTEPH is potentially curable with pulmonary endarterectomy (PEA).^2^ However, diagnosis is often delayed, as non-specific symptoms like dyspnoea overlap with more prevalent conditions such as heart failure with preserved ejection fraction (HFpEF).^3^

While CTEPH develops in up to 4% of patients following an acute pulmonary embolism,^4^ a key diagnostic challenge arises when computed tomography pulmonary angiography (CTPA) fails to show large, organized thrombi. The ventilation-perfusion (V/Q) scan is therefore critical for identifying the characteristic perfusion defects of CTEPH, even when CTPA findings are subtle or absent.^5^ We present the case of a 77-year-old female with HFpEF whose refractory symptoms led to a diagnosis of CTEPH, highlighting the pivotal role of a comprehensive diagnostic approach.

## Summary figure

**Figure:**
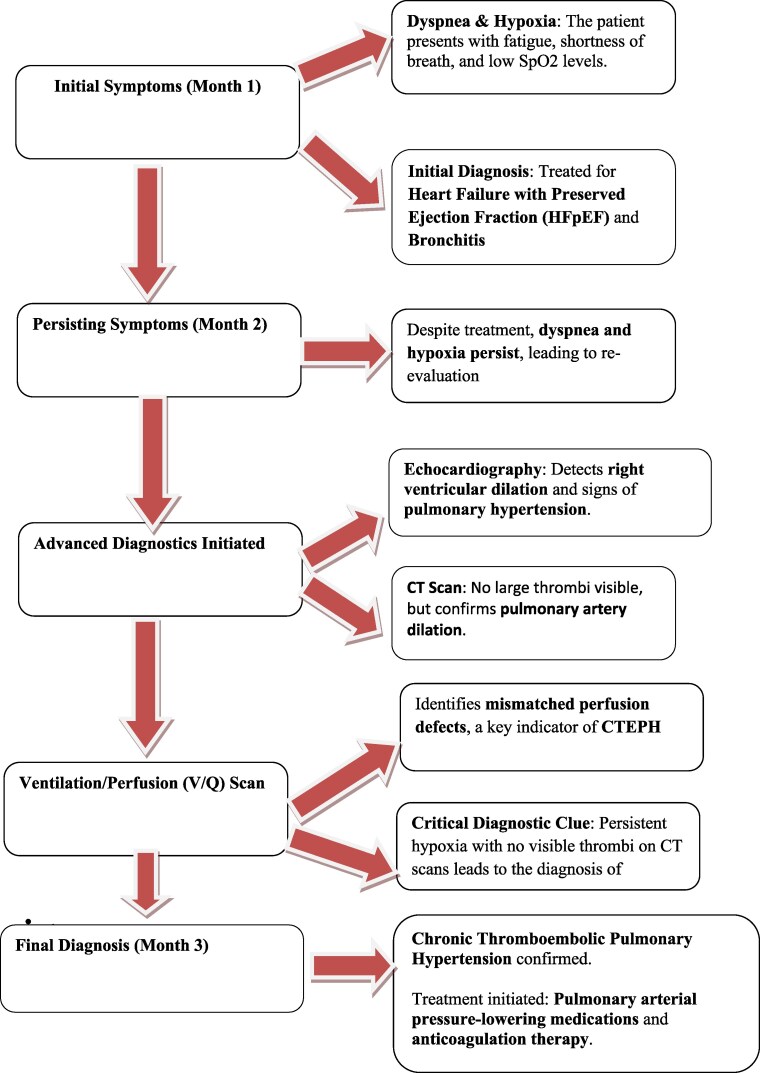


## Case presentation

A 77-year-old female with a two-year history of HFpEF presented with a two-month history of progressive dyspnoea and fatigue. Her home oxygen saturation (SpO_2_) levels were persistently low (80%–84%). She was initially treated for an exacerbation of heart failure, with only temporary improvement. One month later, her symptoms worsened, and on readmission, she had profound dyspnoea despite optimized therapy.

**Figure 1:**
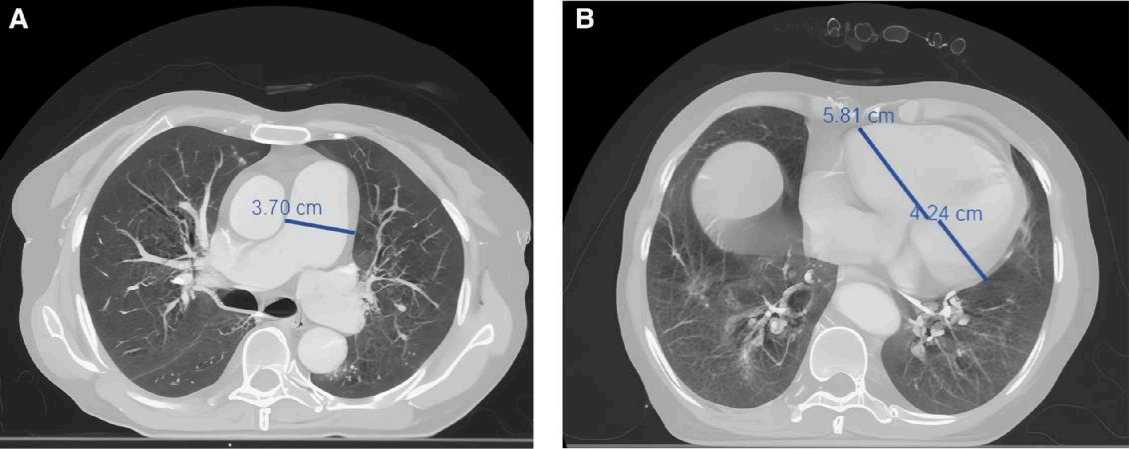
Chest CT scan showing a mobile hyperechoic mass in the right ventricle consistent with thrombus.

**Figure 2:**
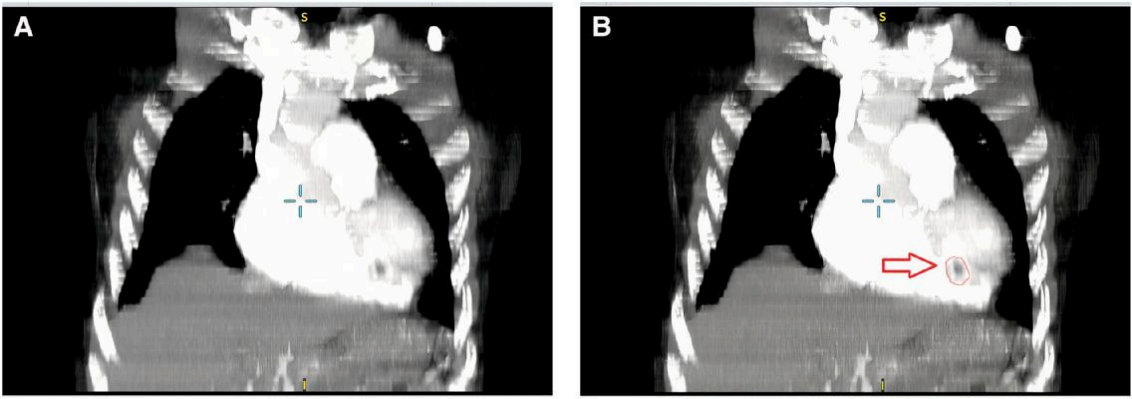
Contrast-enhanced chest CT showing severe right heart strain (RV/LV ratio >1) and dilated pulmonary artery (37 mm).

**Figure 3:**
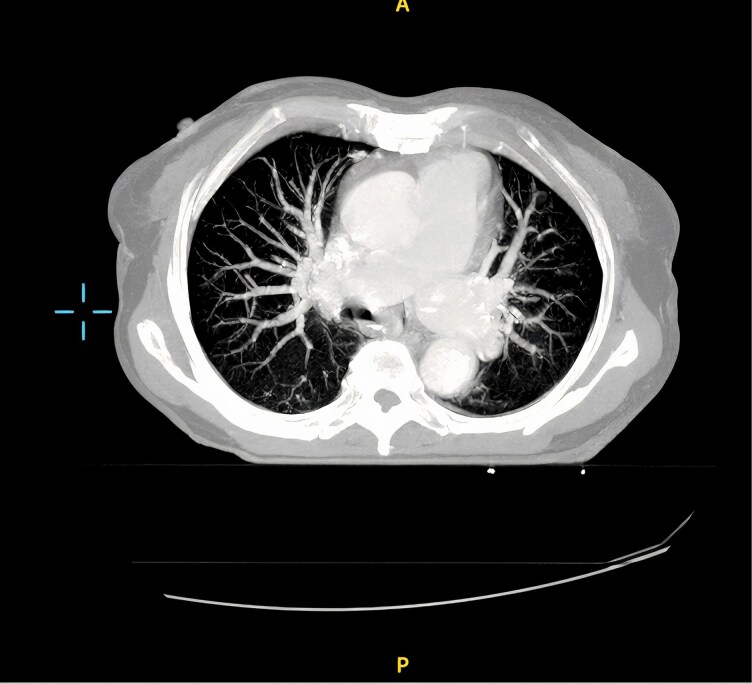
CTPA demonstrating no visible large, organized thrombi in the pulmonary arteries.

**Figure 4:**
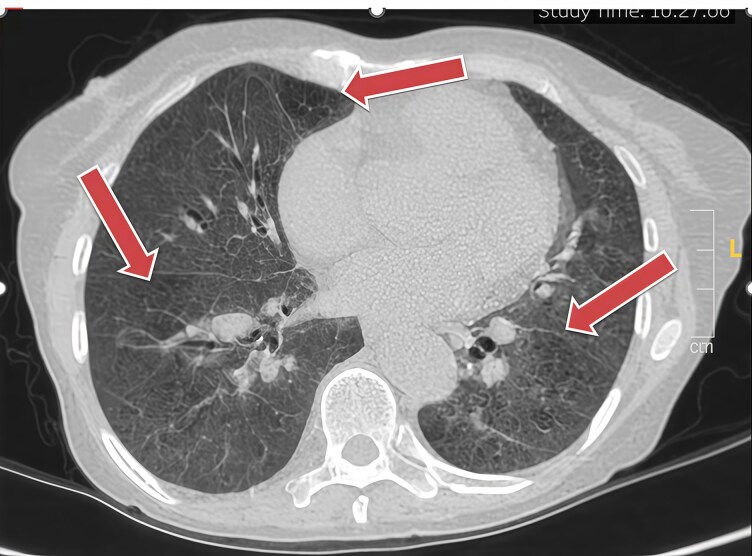
Mosaic attenuation pattern was noted in the lung parenchyma.

An echocardiogram revealed marked right ventricular (RV) dilation and severe pulmonary hypertension, with an estimated pulmonary arterial systolic pressure of 85 mmHg. A mobile, hyperechoic mass (20 × 17 mm) consistent with a thrombus was noted in the right ventricle (*Figure 1*). A contrast-enhanced chest computed tomography (CT) confirmed severe right heart strain (Right Ventricle to Left Ventricle [RV/LV] ratio >1) and a dilated main pulmonary artery (37 mm) (*Figure 2*). Crucially, the CTPA showed no evidence of large, organized thrombi in the main, lobar, or segmental pulmonary arteries (*Figure 3*), though a subtle mosaic attenuation pattern was noted in the lung parenchyma. (*Figure 4*).

Given the presence of the RV thrombus, a comprehensive thrombophilia workup was initiated to evaluate for underlying prothrombotic states. This included testing for antiphospholipid antibodies (lupus anticoagulant, anti-cardiolipin, anti-beta-2 glycoprotein I antibodies), all of which were negative. Age-appropriate malignancy screening was also unremarkable.

Given the strong clinical suspicion for CTEPH despite the negative CTPA, a V/Q scan was performed. The scan was definitive, demonstrating multiple, large, bilateral mismatched segmental perfusion defects—a classic hallmark of CTEPH.

The patient was deemed a high-risk candidate for PEA due to her age and comorbidities. Medical management was initiated with bosentan and apixaban. Her response was remarkable. Dyspnoea improved, exercise tolerance increased, and her supplemental oxygen requirement decreased significantly. At her 3-month follow-up, her resting SpO_2_ was 92%–94%, and a repeat echocardiogram showed a reduction in estimated pulmonary pressure to 70 mmHg.

## Discussion

This case illustrates the critical diagnostic challenges of CTEPH, particularly when confounded by comorbidities like HFpEF. The patient’s persistent and profound hypoxia, disproportionate to her cardiac status, was the key feature that prompted re-evaluation and underscores an important clinical principle: refractory hypoxia should trigger a broader differential diagnosis.

The imaging strategy was central to this diagnosis. While CTPA can be falsely reassuring in the absence of visible endoluminal thrombi, CTEPH is often a disease of organized, wall-adherent material and distal vessel webs that are not well-visualized.^6^ Secondary signs on CT, such as a dilated pulmonary artery and mosaic perfusion, were vital clues that increased suspicion for CTEPH.^7^

In this context, the V/Q scan was invaluable. Its superior sensitivity for detecting perfusion abnormalities makes it the recommended screening test for all patients with unexplained pulmonary hypertension.^5,8^ The starkly positive V/Q scan provided the definitive evidence for diagnosis, validating the diagnostic algorithm recommended by current guidelines.^8^

The management of this case also warrants discussion. The patient was deemed inoperable based on a multidisciplinary assessment of her surgical risk; PEA is the gold standard and first-line treatment for CTEPH when the disease is surgically accessible, particularly in the main, lobar, or segmental arteries.^9^ For patients with inoperable CTEPH, that deemed inoperable or with persistent/recurrent pulmonary hypertension after PEA, alternatives include balloon pulmonary angioplasty (BPA) and targeted medical therapy (e.g. riociguat), but BPA is also not well-experienced in our centre, so medical therapy is the cornerstone of treatment. While riociguat, a soluble guanylate cyclase stimulator, is the first-line approved medical therapy with proven efficacy,^8^ the decision was made in this case to initiate bosentan, an endothelin receptor antagonist. This choice was based on institutional experience and immediate availability at the time of diagnosis. The patient’s significant and rapid clinical improvement on this regimen, along with anticoagulation, supported its continuation. The presence of an RV thrombus further reinforced the absolute necessity of lifelong anticoagulation to prevent further thrombotic events.^10^ This outcome emphasizes that even when PEA is not an option, a correct diagnosis is crucial for initiating targeted medical therapies that can dramatically improve quality of life.^11^

## Conclusion

CTEPH can be a hidden cause of refractory dyspnoea and hypoxia, particularly in patients with pre-existing cardiopulmonary disease. Clinicians must maintain a high index of suspicion and not be dissuaded by a CTPA that lacks visible thrombi. The diagnostic pathway should include a V/Q scan, which remains the most sensitive non-invasive test. Early and accurate diagnosis is essential to initiating life-improving therapy and preventing the irreversible consequences of this challenging but treatable condition.

## Lead author biography


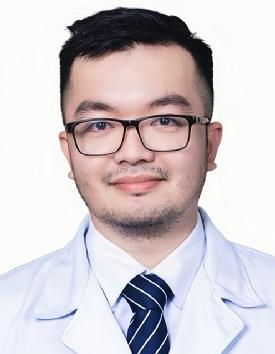


Hoang Phu Quy, MD, is an Internal Resident Doctor at VinUniversity, Hanoi, Vietnam. He is dedicated to advancing knowledge in cardiovascular medicine, with a special interest in diagnostic challenges and complex conditions such as chronic thromboembolic pulmonary hypertension (CTEPH). Dr. Quy is passionate about improving patient care through thorough clinical evaluations and innovative diagnostic approaches. He is currently engaged in multiple case studies and research projects aimed at enhancing treatment outcomes for patients with cardiopulmonary diseases. Dr. Quy’s work reflects his commitment to excellence in both clinical practice and research.

## Data Availability

The data underlying this article are available in the article. Further inquiries can be directed to the corresponding author.

## References

[1] Otani N, Watanabe R, Tomoe T, Sato H, Kondo Y, Nakajima K, et al Pathophysiology and treatment of chronic thromboembolic pulmonary hypertension. Int J Mol Sci 2023;24:3979. 36835383 10.3390/ijms24043979 PMC9968103

[2] Bishay O, Alshammari F, Yamada T, Singh H, Zhou L, Patel A, et al Pulmonary endarterectomy: the potentially curative treatment for patients with chronic thromboembolic pulmonary hypertension. J Thorac Dis 2022;14:3038–3051.

[3] Khan SS, Kalhan R. Comorbid chronic obstructive pulmonary disease and heart failure: shared risk factors and opportunities to improve outcomes. Ann Am Thorac Soc 2022;19:897–899. 35648080 10.1513/AnnalsATS.202202-152ED PMC9169135

[4] Ende-Verhaar MM, Cannegieter SC, Vonk Noordegraaf A, Delcroix M, Pruszczyk P, Mairuhu ATA, et al Incidence of chronic thromboembolic pulmonary hypertension after acute pulmonary embolism: a contemporary view of the published literature. Eur Respir J 2014;43:1363–1370. 10.1183/13993003.01792-2016 28232411

[5] Fathala A, Aldurabi A. Frequency of computed tomography abnormalities in patients with chronic thromboembolic pulmonary hypertension: a comparative study between lung perfusion scan and computed tomography pulmonary angiography. Multidiscip Respir Med 2021;16:753. 34322231 10.4081/mrm.2021.753 PMC8273626

[6] Gopalan D, Delcroix M, Held M. Diagnosis of chronic thromboembolic pulmonary hypertension. Eur Respir Rev 2017;26:160108. 28298387 10.1183/16000617.0108-2016 PMC9488918

[7] Ristagno RL, Zhang J, Patel N, Gupta S, Nair A, Krishnan S, et al Imaging of chronic thromboembolic pulmonary hypertension: a state-of-the-art review. J Am Heart Assoc 2023;12:e029322.

[8] Humbert M, Kovacs G, Hoeper MM, Badagliacca R, Berger RMF, Brida M, et al 2022 ESC/ERS guidelines for the diagnosis and treatment of pulmonary hypertension. Eur Heart J 2022;43:3618–3731. 36017548 10.1093/eurheartj/ehac237

[9] Quadery SR, Swift AJ, Billings CG, Thompson AAR, Elliot CA, Charalampopoulos A, et al The impact of patient choice on survival in chronic thromboembolic pulmonary hypertension. Eur Respir J 2018;52:1800589. 30002102 10.1183/13993003.00589-2018 PMC6340636

[10] Medrek S, Safdar Z. Epidemiology and pathophysiology of chronic thromboembolic pulmonary hypertension: risk factors and mechanisms. Methodist Debakey Cardiovasc J 2016;12:195–198. 28289493 10.14797/mdcj-12-4-195 PMC5344468

[11] Delcroix M, Lang I, Pepke-Zaba J, Jansa P, D’Armini AM, Snijder R, et al Long-term outcome of patients with chronic thromboembolic pulmonary hypertension: results from an international prospective registry. Circulation 2016;133:859–871. 26826181 10.1161/CIRCULATIONAHA.115.016522

